# Map Archive Mining: Visual-Analytical Approaches to Explore Large Historical Map Collections

**DOI:** 10.3390/ijgi7040148

**Published:** 2018-04-13

**Authors:** Johannes H. Uhl, Stefan Leyk, Yao-Yi Chiang, Weiwei Duan, Craig A. Knoblock

**Affiliations:** 1 Department of Geography, University of Colorado Boulder, Boulder, CO 80309, USA; stefan.leyk@colorado.edu; 2 Spatial Sciences Institute, University of Southern California, Los Angeles, CA 90089, USA; yaoyic@usc.edu (Y.-Y.C.); weiweidu@usc.edu (W.D.); knoblock@isi.edu (C.A.K.)

**Keywords:** map processing, retrospective landscape analysis, visual data mining, image information mining, low-level image descriptors, color moments, t-distributed stochastic neighborhood embedding, USGS topographic maps, Sanborn fire insurance maps

## Abstract

Historical maps are unique sources of retrospective geographical information. Recently, several map archives containing map series covering large spatial and temporal extents have been systematically scanned and made available to the public. The geographical information contained in such data archives makes it possible to extend geospatial analysis retrospectively beyond the era of digital cartography. However, given the large data volumes of such archives (e.g., more than 200,000 map sheets in the United States Geological Survey topographic map archive) and the low graphical quality of older, manually-produced map sheets, the process to extract geographical information from these map archives needs to be automated to the highest degree possible. To understand the potential challenges (e.g., salient map characteristics and data quality variations) in automating large-scale information extraction tasks for map archives, it is useful to efficiently assess spatio-temporal coverage, approximate map content, and spatial accuracy of georeferenced map sheets at different map scales. Such preliminary analytical steps are often neglected or ignored in the map processing literature but represent critical phases that lay the foundation for any subsequent computational processes including recognition. Exemplified for the United States Geological Survey topographic map and the Sanborn fire insurance map archives, we demonstrate how such preliminary analyses can be systematically conducted using traditional analytical and cartographic techniques, as well as visual-analytical data mining tools originating from machine learning and data science.

## 1. Introduction

Historical maps contain valuable information about the Earth’s surface in the past. This information can provide a detailed understanding of the evolution of the landscape, as well as the interrelationships between human-made structures (e.g., transportation networks, settlements), vegetated land cover (e.g., forests, grasslands), terrain, and hydrographic features (e.g., stream networks, water bodies). However, this spatial information is typically locked in scanned map images and needs to be extracted to gain access to the geographic features of interest in machine readable data formats that can be imported into geospatial analysis environments.

**Figure 1.**
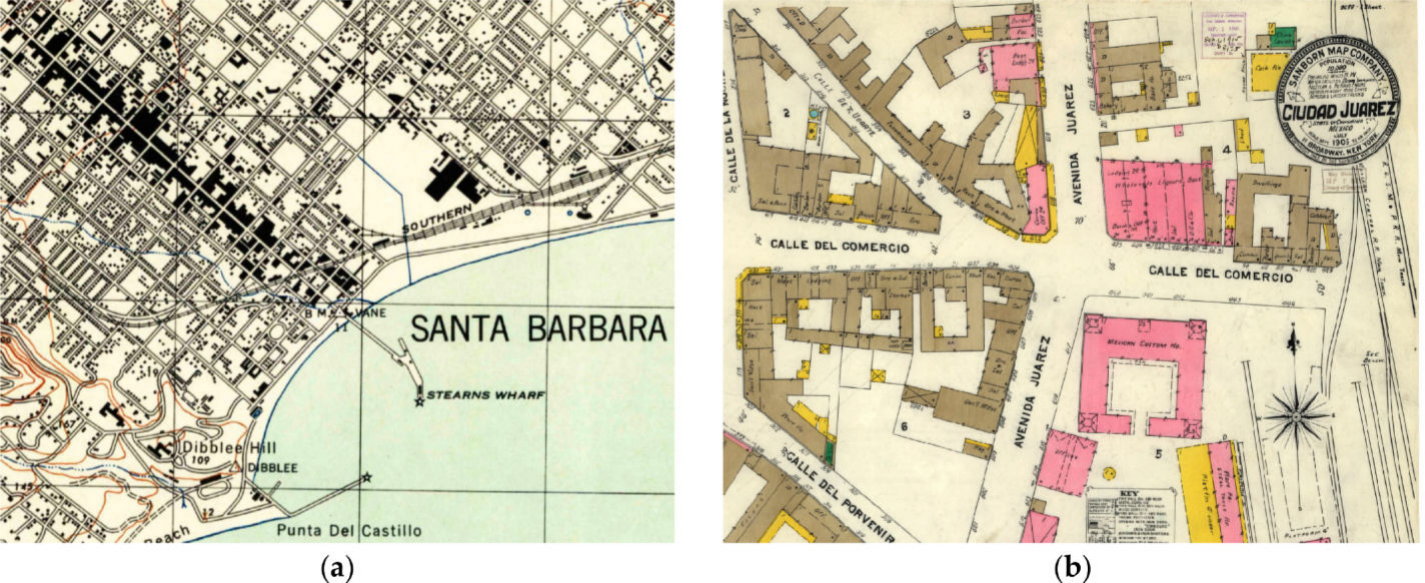
Examples of historical map documents: (**a**) Subsection of a USGS topographic map 1:31,680 of Santa Barbara (California, 1944) and (**b**) the Sanborn fire insurance map of the city center of Ciudad Juárez (Mexico, 1905).

Several efforts have recently been conducted in different countries to systematically scan, georeference, and publish entire series of topographic and other map documents. These developments include efforts at the United States Geological Survey (USGS), that scanned and georeferenced approx. 200,000 topographic maps published between 1884 and 2006 at different cartographic scales between 1:24,000 and 1:250,000 [1] and the Sanborn fire insurance map collection maintained by the U.S. Library of Congress, which contains approximately 700,000 sheets of large-scale maps of approximately 12,000 cities and towns in the U.S., Canada, Mexico, and Cuba, out of which approximately 25,000 map sheets from over 3000 cities have been published as scanned map documents [2–4] (Figure 1). Furthermore, the National Library of Scotland scanned and georeferenced more than 200,000 topographic map sheets and town plans for the United Kingdom dating back to the 1840s and provides many of them as seamless georeferenced raster layers [5,6].

These developments, alongside with advances in information extraction and the processing, storage and distribution of large data volumes, offer great potential for automated, large-scale information extraction from historical cartographic document collections in order to preserve the contained geographic information and make it accessible for geospatial analysis. Due to the large amount of data contained in these map archives, information extraction has to achieve high degrees of automation. For example, the USGS map archive has an approximate uncompressed data volume of 50 terabytes, whereas the data volume of currently digitally-available Sanborn fire insurance map sheets can be estimated to approximately 3.7 terabytes.

**Figure 2.**
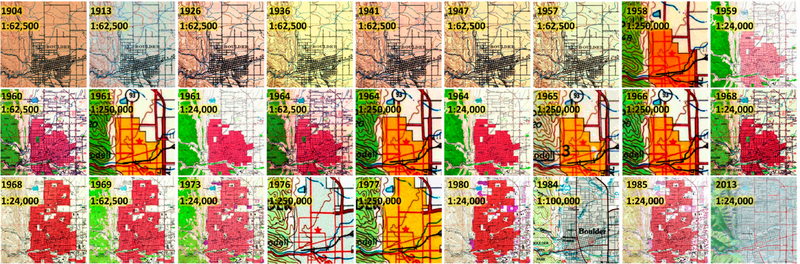
Available USGS topographic map sheets covering Boulder, Colorado (USA) from 1904 to 2013 at various map scales.

This constitutes a challenging task given the high variability in the content and quality of map sheets within an archive. Possible reasons for such variability are different conditions of the archived analogue map documents, differences in the scan quality, as well as changes in the best practices in cartographic design that may have resulted in different symbologies across map editions (Figure 2).

Typically, knowledge about the variability in content and quality of map archives are a priori not available, since such large amounts of data cannot be analyzed manually. However, such information is critical for a better understanding of the data sources and the design of efficient and effective information extraction methods. Thus, there is an urgent demand to develop a systematic approach to explore such digital map archives, efficiently, prior to the actual extraction process, similar to existing efforts for remote sensing data. In this contribution, we examine various techniques that could be used to build an image information mining system for digital cartographic document archives in combination with metadata analysis. These techniques aim to answer the following questions a potential user of such map archives may ask prior to the design and implementation of information extraction methods:

- **What is the spatial and temporal coverage of the map archive content and does it vary across different cartographic scales?** The user will need to know the potential extent, temporally and spatially, of the extracted data to understand benefit and value of the intended information extraction effort and for comparing different map archives.
- **How accurate is the georeference of maps contained in the archive? Does the accuracy vary in the spatio-temporal domain?** This constitutes a pressing question if ancillary geospatial data is used for the information extraction and certain degrees of spatial alignment with map features are required. For example, if it is possible to a priori identify map sheets likely to suffer from a high degree of positional inaccuracy, the user can exclude those map sheets from template or training data collection and, thus, reduce the amount of noise in the collected training data.
- **How much variability is there in the map content, regarding color, hue, contrast, and in the cartographic styles used to represent the symbol of interest?** This is a central question affecting the choice and design of a suitable recognition model. More powerful models or even different models for certain types of maps may be required if the representation of map content of interest varies heavily across the map archive. Furthermore, knowledge of variations in map content and similarity between individual map sheets is useful to optimize the design of training data sampling and to ensure the collection of representative and balanced training samples.

The set of methods described herein help determine the spatial-temporal coverage of a historical map archive, its content, existing variations in cartographic design, and to partially assess the spatial accuracy of the maps, which are all critical aspects for information extraction. These preprocessing stages are often neglected in published research that traditionally focuses on the extraction methods. The presented approaches range from pure metadata analysis to descriptor-based visual data mining techniques, such as image information mining [7] used for the exploration of large remote sensing data archives. These methods are exemplified using the USGS topographic map archive and the Sanborn fire insurance map collection.

Section 2 gives an overview of related research. Section 3 introduces the data used in this work, and Chapter 4 describes the methods. Section 5 presents and discusses the results, and Section 6 contains some concluding remarks and directions for future research.

## 2. Background and Related Research

### 2.1. Map Processing

Map processing, or information extraction from digital map documents, is a branch of document analysis that focuses on the development of methods for the extraction and recognition of information in scanned cartographic documents. Map processing is an interdisciplinary field that combines elements of computer vision, pattern recognition, geomatics, cartography, and machine learning. The main goal of map processing is to “unlock” relevant information from scanned map documents to provide this information in digital, machine-readable geospatial data formats as a means to preserve the information digitally and facilitate the use of these data for analytical purposes [8].

Remotely-sensed Earth observation data from space and airborne sensors has been systematically acquired since the early 1970s and provides abundant information for the monitoring and assessment of geographic processes and how they interact over time. However, for the time periods prior to operational remote sensing technology, there is little (digital) information that can be used to document these processes. Map processing often focuses on the development of information extraction methods from map documents or engineering drawings created prior to the era of remote sensing and digital cartography, thus expanding the temporal extent for carrying out geographic analyses and landscape assessments to more than 100 years in many countries.

Information extraction from map documents includes the steps of *recognition* (i.e., identifying objects in a scanned map such as groups of contiguous pixels with homogeneous semantic meaning), and *extraction* i.e., transferring these objects into a machine-readable format (e.g., through vectorization). Extraction processes typically involve image segmentation techniques based on histogram analysis, color-space clustering, region growing or edge detection. Recognition in map processing is typically conducted using computer vision techniques, including template matching techniques involving feature (e.g., shape) descriptors, cross-correlation measures, etc. Exemplary applications of map processing techniques include the extraction of buildings [9–11], road networks [12], contour lines [13], composite forest symbols [14], and the recognition of text from map documents [15,16]. Most approaches rely on handcrafted or manually collected templates of the cartographic symbol of interest and involve a significant level of user interaction, which impedes the application of such methods for large-scale information extraction tasks where high degrees of automation are necessary to process documents with high levels of variation in data quality.

### 2.2. Recent Developments in Map-Based Information Extraction

The availability of abundant contemporary geospatial data for many regions of the world offers new opportunities to employ them as ancillary information to facilitate the extraction and analysis of geographic content from historical map documents. This includes the use of contemporary spatial data for georeferencing historical maps [17], assessing the presence of objects in historical maps across time [18], or the automated collection of template graphics for cartographic symbols of interest [19].

Most existing approaches for content extraction from historical maps still require a certain degree of user interaction to ensure acceptable extraction performance for individual map sheets, e.g., [20]. To overcome this persistent limitation, Refs. [21,22] propose the use of active learning and similar interactive concepts for more efficient recognition of cartographic symbols in historical maps, whereas [23] examine the usefulness of crowd-sourcing for the same purpose.

Moreover, the recent developments in deep machine learning in computer vision and image recognition have catalyzed the use of such techniques for geospatial information extraction from Earth observation data [24–33]. This methodological development naturally projects into the idea of applying state-of-the-art machine learning techniques for information extraction from scanned cartographic documents, despite their fundamentally different characteristics compared to remotely-sensed data. Key in both cases is the need for abundant and representative training data which requires automated sampling techniques. First attempts in this direction have used ancillary geospatial data for the collection of large amounts of training data in historical maps [34–36].

Alongside with the increasing availability of whole map archives as digital data, central challenges in map processing include the handling of the sheer data volume, the differences in cartographic scales and designs, changes in content, graphical quality, and cartographic representations, the spatial and temporal coverage of the map sheets, and the spatial accuracy of the georeferenced map which dictates the degree of spatial agreement to contemporary geospatial ancillary data. While the previously described approaches represent promising directions towards higher levels of automation, they imply that the graphical characteristics of the map content to be extracted are known and that map scale and cartographic design remain approximately the same across the processed map documents.

### 2.3. Image Information Mining

The remote sensing community faces similar challenges. The steadily increasing amount of remotely-sensed Earth observation data requires effective mining techniques to explore the content of large remote sensing data archives. Therefore, visual data mining techniques have successfully been used to comprehensively visualize the content of such archives. Such image information mining systems facilitate discovery and retrieval using available metadata, and they make use of the similarity of the content of the individual datasets, or of patches of these [37,38], and guide exploratory analysis of large amounts of data to support subsequent development of information extraction methods. Such a system has, for example, been implemented for TerraSAR-X data [39], or for patches of Landsat ETM+ data and the UC Merced benchmark dataset [40]. These systems are based on spectral and textural descriptors precomputed at dataset or patch level that are then combined to multidimensional descriptors characterizing spectral-textural content of the datasets or patches. Other approaches include image segmentation methods to derive shape descriptors [41], integrate spatial relationships between images into the image information mining system [42], or make use of structural descriptors to characterize the change of geometric patterns over time across datasets within remote sensing data archives [43]. Comparison of these descriptors facilitates the retrieval of similar content across large archives. These approaches include methods for dimensionality reduction to visualize an entire data archive in a two or three-dimensional feature space based on content similarity.

Whereas in remote sensing data archives the spatio-temporal coverage of the data and their quality is relatively well-known based on the sensor characteristics (e.g., the time of operationality, satellite orbit, revisiting frequency, knowledge about physical parameters affecting data quality), this may not always be the case for historical map archives, where metadata on spatial-temporal data coverage might not be available or available in semi-structured data formats only, impeding direct and systematic analysis.

## 3. Data

In this study, we analyzed map documents from the USGS map archive for the states of California (14,831 map sheets) and Colorado (6964 map sheets). These map sheets were scanned by the USGS at a resolution of approximately 500 dpi (dots per inch) resulting in TIF files with an uncompressed data volume of more than 5.3 terabytes for the two states under study. Whereas the authors were granted access to these data covering the two states at the original scanning resolution, slightly downsampled versions of these map documents covering the whole U.S. can be publicly accessed at [44].

The delivered raw data was not georeferenced, but included metadata for the georeferencing process, i.e., coordinate pairs and error estimates of the ground control points (GCP) used for each individual map sheet allowing for batch georeferencing of the map sheets on the user side. In addition to that, corner coordinates of each map sheet are reported in the metadata allowing for the creation of spatial footprints (i.e., the USGS map quadrangle outlines) without georeferencing them. These metadata were available in a structured form in XML or CSV formats.

Furthermore, we used metadata of the Sanborn fire insurance map archive in this study, including the locations (i.e., geographic names), the reference years, and the number of map sheets available for each location, which is available as semi-structured HTML web content from the U.S. Library of Congress website [45].

## 4. Methods

**Figure 3.**
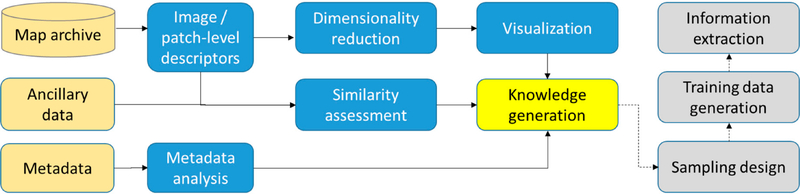
The methodology for metadata analysis of and content-based knowledge generation from map archives to facilitate information extraction.

We conducted ***metadata analysis*** for the USGS topographic map archive exemplified for the states of California and Colorado based on structured metadata, as well as for the Sanborn fire insurance map archive in the United States based on semi-structured metadata. Next, we carried out ***content-based image analysis*** for the USGS topographic map archive covering the state of Colorado at different map scales, involving the use of image descriptors, dimensionality reduction, and data visualization methods, as well as a similarity assessment based on geospatial ancillary data. The workflow diagram in Figure 3 shows how the proposed methods (in blue) based on given map data, metadata, and ancillary data (in beige) can be incorporated to generate knowledge useful for subsequent information extraction procedures (in grey).

### 4.1. Metadata Analysis

#### 4.1.1. Spatio-Temporal Coverage Analysis

Based on the ***structured*** metadata (i.e., map scale, reference year, corner coordinates, and GCP coordinate pairs in XML and CSV data formats) available for the USGS map archive, we created several aspatial visualizations (i.e., histograms and violin plots) illustrating the spatio-temporal coverage of the map archive. Based on the spatial footprints of the map sheets, we computed statistical measures, such as the earliest reference year per map quadrangle, and visualized them, spatially, in order to reveal potential spatial patterns of the coverage in the spatio-temporal domain (Section 5.1.1).

We retrieved the ***semi-structured*** metadata of the Sanborn map archive from HTML-based web content to derive the geospatial location of each map location (i.e., town or city name, county, and state) using web-based geocoding services to then visualize data availability and spatio-temporal coverage of Sanborn map documents (Section 5.1.1).

#### 4.1.2. Assessing Positional Accuracy

Positional accuracy of scanned maps can be caused by several factors, such as paper map distortions due to heat or humidity, the quality of surveying measurements on which the map production is based, deviations from the local geodetic datum at data acquisition time, cartographic generalization, and distortions introduced during the scanning and georeferencing process. While most of these effects cannot be reconstructed or quantified in detail, metadata delivered with the USGS topographic map archive contains information about the GCPs used for georeferencing the scanned map documents that we used for a partial assessment of these distortions and resulting positional inaccuracies.

The USGS topographic map quadrangle boundaries represent a graticule. For example, the corner coordinates for quadrangles of scale 1:24,000 are spaced in a regular grid of 7.5’ × 7.5’. Additionally, a finer graticule of 2.5’ × 2.5’ is depicted in the maps. The intersections of this fine graticule are used by the USGS to georeference the maps. Therefore, we collected the pixel coordinates at those locations (i.e., the GCPs), and used the corresponding known world coordinates of the graticule intersections to establish a second-order polynomial transformation based on least-squares adjustment. We used this transformation to warp the scanned document into a georeferenced raster dataset. We reported the GCP coordinate pairs in the metadata, as well as an error estimate per GCP that provides information on the georeference accuracy in pixels. Based on these error estimates given in pixel units and the spatial resolution of the georeferenced raster given in meters, we calculated the root mean standard error (RMSE) reflecting the georeference accuracy in meters. We appended these RMSE values as attributes to the map quadrangle polygons to visualize the georeference accuracy across the spatial-temporal domain.

Furthermore, we characterized the distortion introduced to the map by the warping process using displacement vectors computed between the known world coordinates of each GCP (i.e., the graticule intersections) and the world coordinates corresponding to the respective pixel coordinates after applying the second-order polynomial transformation. These displacement vectors reflected geometric distortions and positional inaccuracy in the original map (i.e., prior to the georeferencing process) but are also affected by additional distortions introduced during georeferencing or through scanner miscalibration.

Assuming that objects in the map are affected by the same degree of inaccuracy like the graticule intersections, the magnitudes of these displacement vectors make it possible to estimate the maximum displacements to be expected between objects in the map and their real-world counterparts that may not be corrected by the second-order polynomial transformation. We visualized these displacement vectors to indicate the magnitude and direction of such distortions, and potentially identify anomalies (Section 5.1.2).

### 4.2. Content-Based Image Analysis

The presented metadata-based analysis provides valuable insights of spatial-temporal map availability, coverage, and spatial accuracy without analyzing the actual content of the map archives. However, it is important to inform the analyst about the degree of heterogeneity at the content-level. Therefore we computed low-level image descriptors (i.e., color moments) at multiple levels of granularity, i.e., for individual map sheets and for patches of maps. We then use these image descriptors as the input to a dimensionality reduction method (i.e., t-distributed stochastic neighborhood embedding) in order to visualize the maps or map patches in a two or three dimensional space for effective visual map content assessment, and analytical assessment of their similarity.

#### 4.2.1. Low-Level Image Descriptors

In order to obtain detailed knowledge about the content of map archives, we developed a framework based on low-level image descriptors computed for each map or map patch. We employed color-histogram based moments (i.e., mean, standard deviation, skewness, and kurtosis, see [46]) computed for each image channel in the RGB color space. Mean and standard deviation characterize hue, brightness, and contrast levels of an image, skewness and kurtosis indicate the symmetry and flatness of the probability density of the color distributions, and thus reflect color spread and variability of an image. They are invariant to rotations, however, they do not take into account textural information contained in the image. We computed these four measures for each channel of an image and stacked them together to a 12-dimensional feature descriptor, at the image or patch level. In the case of scanned map documents, such descriptors make it possible to retrieve maps or patches of maps of similar background color (depending on paper type and scan contrast level), and maps of similar dominant map content, such as water bodies, urban areas, or forest cover. This similarity assessment was based on distances in the descriptor feature space and could also involve metadata (e.g., map reference year), or ancillary geospatial data, to assess map content similarity across or within different geographic settings.

#### 4.2.2. Dimensionality Reduction

Furthermore, we employed approaches for dimensionality reduction such as t-distributed stochastic neighborhood embedding (t-SNE, [47]) to visualize the image data based on the similarity in feature space. T-SNE allows for reducing the dimensionality of high-dimensional data, where the relative distances between the data points in the reduced feature space reflect the similarity of the data points in the original feature space. T-SNE is based on pair-wise similarities of data points, where the corresponding similarity measures in the target space are modelled by a Student’s t distribution [48]. The transformation of the data points into the target space of dimension 2 or 3 is conducted in an iterative optimization process that aims to reflect local similarity and global clustering effects of the original space in the target space of a reduced dimensionality. This iterative process uses a gradient descent method to iteratively minimize a cost function and can be controlled by several user-defined parameters, such as the learning rate, perplexity, and maximum number of iterations. T-SNE is able to create visually-appealing data representations in two- or three-dimensional spaces reflecting the inherent similarity and variability of the data, but may be prone to non-convergence effects resulting in meaningless visualizations if the chosen optimization parameters are not suitable for the data used. For the t-SNE transformations described in this work, we used a perplexity value of 30, a learning rate of 200, and a maximum number of 1000 iterations, in order to yield visually-satisfactory results, i.e., showing meaningful spatial patterns, such as clusters. The application of this method to image-moments-based map descriptors facilitates the visual or quantitative identification of clusters of similar map sheets and provides a better understanding of the content of large map archives and their inherent variability. This kind of similarity assessment and metadata analysis is useful in generating knowledge which can be used to guide sampling designs to generate template or training data for supervised information extraction techniques.

#### 4.2.3. Multi-Level Content Analysis

We computed image descriptors at different levels of spatial granularity, at the map level and the map patch level.

##### Content analysis at map level:

We analyzed the content of the entire map archive with respect to similarities between the individual map sheets by computing the image-moments-based map descriptors and transforming them into a three-dimensional space using t-SNE that can be visualized and interpreted intuitively.

##### Content analysis at map patch level:

Map patches can be compared within a single map sheet, or across multiple map sheets. In order to assess the content ***within map sheets***, we partitioned the map documents into tiles of a fixed size. We used the quadrangle boundaries based on corner coordinates delivered in the metadata to clip the map contents and removed non-geographic content in the map sheet edges. Then, we computed low-level descriptors based on color moments for each individual patch. If the patch size was chosen small enough, it appeared computationally feasible to use the raw (or down-sampled) patch data (e.g., a line vector of all pixel values in the patch) as a basis for t-SNE transformations. This could be useful if one desires to introduce a higher degree of spatiality, and even directionality, when assessing the similarity between the patches.

If variations of specific cartographic symbols ***across map sheets*** are of interest and have to be characterized, ancillary geospatial data can be employed to label the created map patches based on their spatial relationships to the ancillary data. For example, it may be important to assess the differences in cartographic representations of dense urban settlement areas across map sheets, in order to design a recognition model for urban settlement. To test such a situation, we employed building footprint data with built-year information and the respective spatio-temporal coverage to reconstruct settlement distributions in a given map reference year (see [49]). Based on these reference locations, we then computed building density surfaces for each map reference year and used appropriate thresholding to approximately delineate dense settlement areas for a given point in time. Based on spatial overlap between map patches and these dense reference settlement areas, we were able to identify map patches that are likely to contain urban area symbols across multiple maps. We then visualized these selected map patches in an integrated manner using t-SNE arrangements.

## 5. Results

### 5.1. Metadata Analysis

#### 5.1.1. Metadata-Based Spatial-Temporal Coverage Analysis

**Figure 4.**
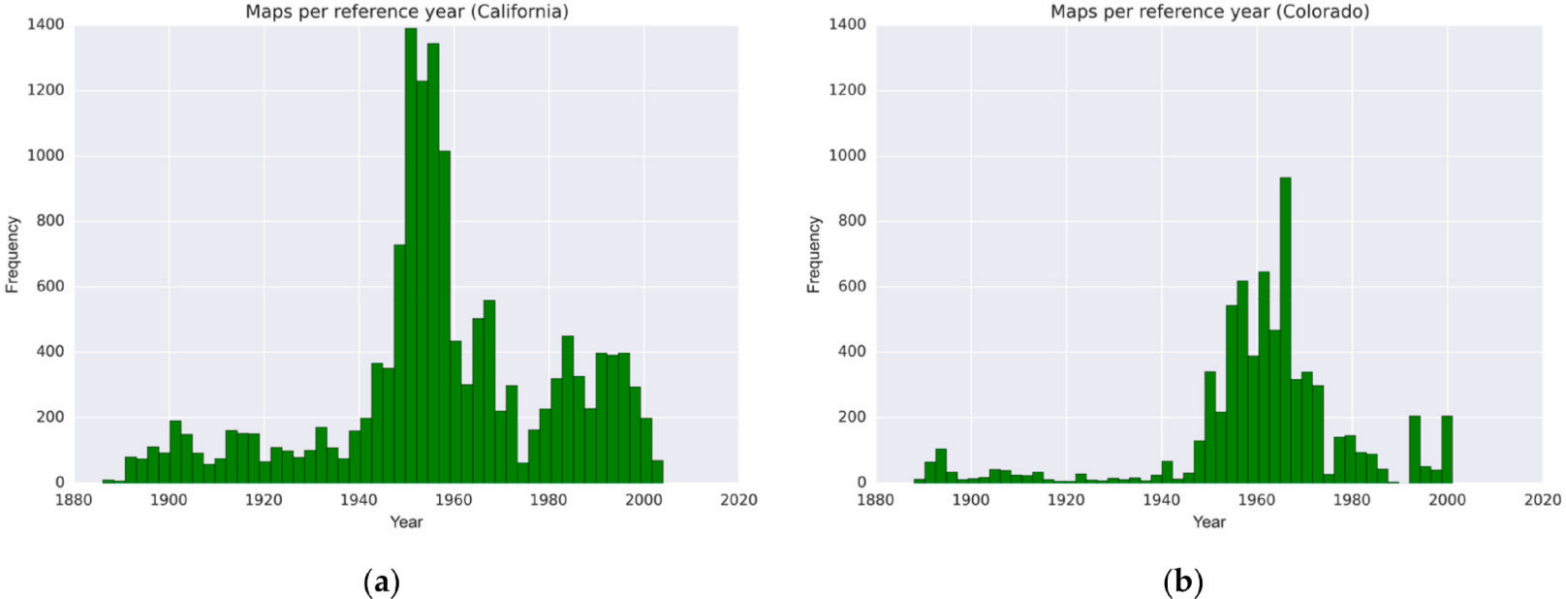
Histograms of USGS topographic maps (all available map scales) by reference year, (**a**) in California, and (**b**) in Colorado (USA).

First, we analyzed the temporal coverage of the map archives. For the USGS map archive, we created histograms based on the map reference year included in the accompanying metadata (Figure 4). It can be seen that the peak of map production in California was in the 1950s, and slightly later, in the 1960s in Colorado.

**Figure 5.**
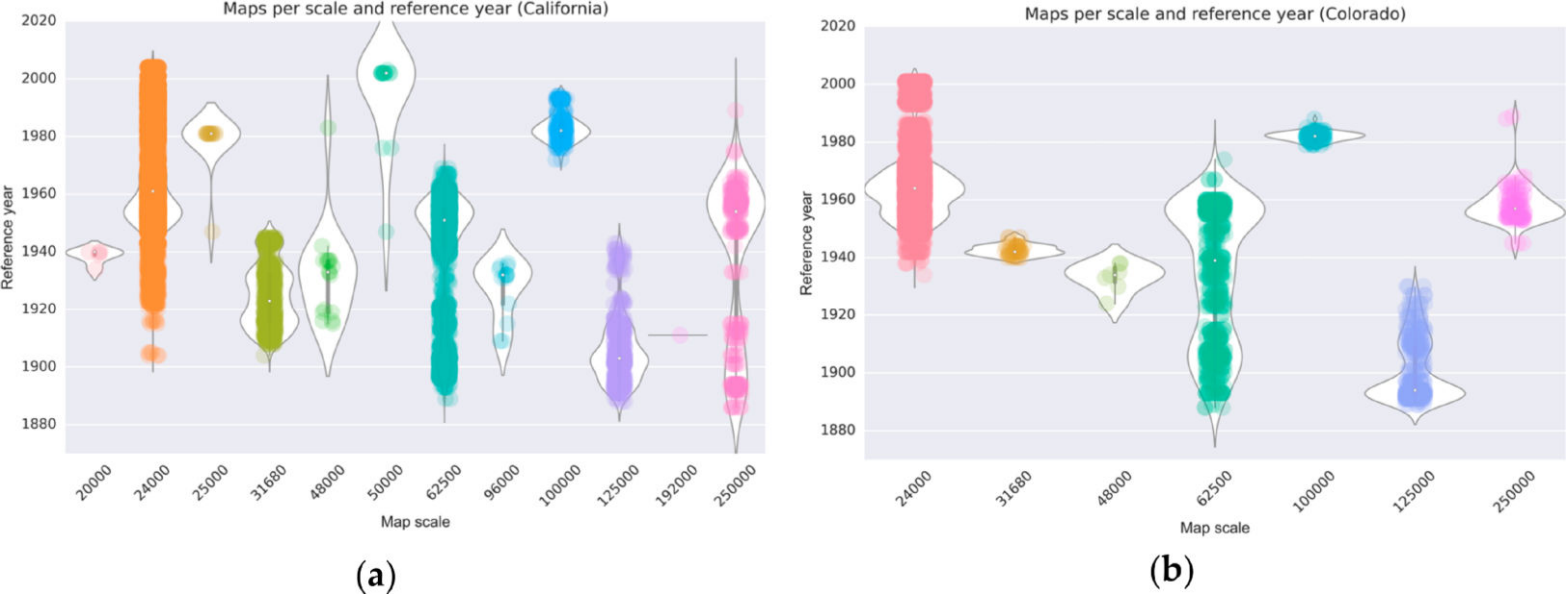
Produced USGS topographic maps per reference year and map scale (**a**) in California, and (**b**) in Colorado (USA).

In addition to that, we assessed map production activity over time for different strata of map scales shown for the states of California and Colorado (Figure 5). These plots show the temporal distribution of published map editions (represented by the dots) and give an estimate of the underlying probability density (represented by the white areas) that indicates the map production intensity over time, separate and relative for each map scale. For example, this probability density estimate reveals a peak of map production at scale 1:62,500 in Colorado (Figure 5b) around 1955 which is not visible in scatterplot alone. Such a representation helps to understand which time span can be covered with maps of various scales and, thus, can be used to determine which products to focus on for a particular purpose. This is important because maps of different scales contain different levels of detail resulting from cartographic generalization.

**Figure 6.**
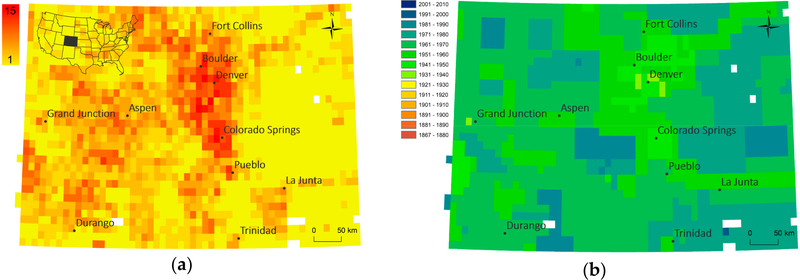
(**a**) Map edition counts and (**b**) earliest map production year per 1:24,000 map quadrangle in the state of Colorado (USA) based on metadata analysis.

**Figure 7.**
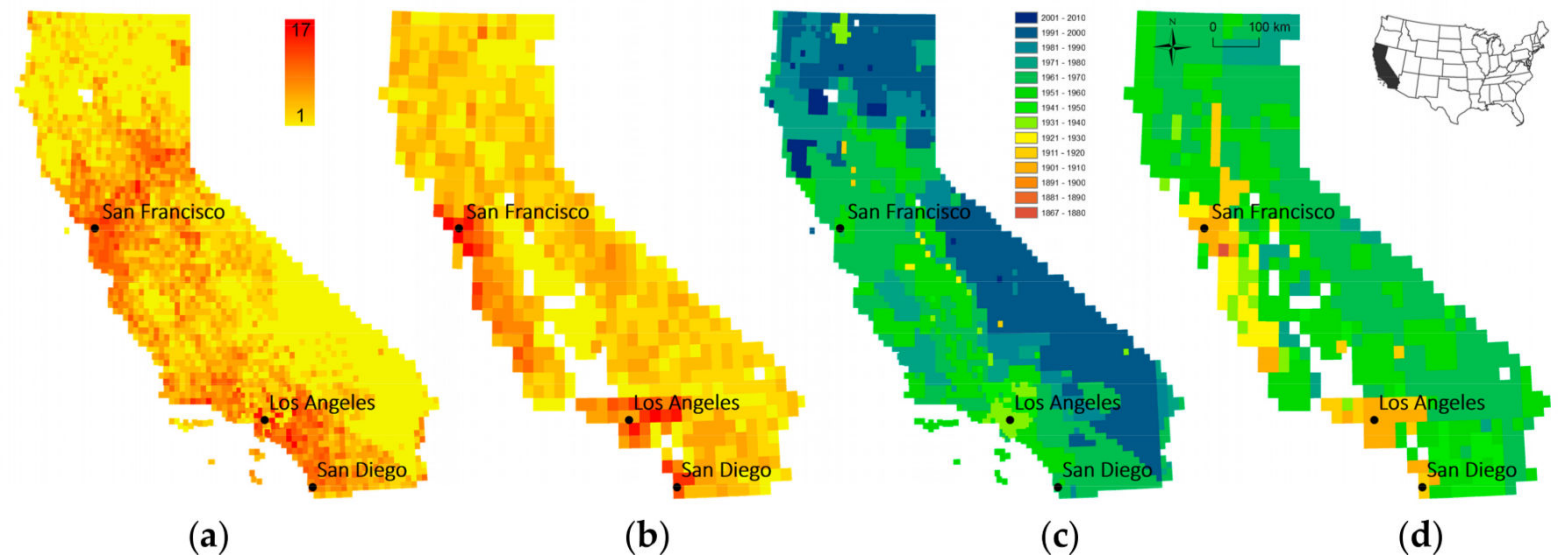
(**a**) Map edition counts per 1:24,000 map quadrangle, (**b**) map edition counts per 1:62,500 map quadrangle, (**c**) earliest map production year per 1:24,000 map quadrangle, and (**d**) earliest map production year per 1:62,500 map quadrangle in the state of California (USA) based on metadata analysis.

In order to assess the spatial variability of map availability in a map archive over time, we visualized the number of map editions and the earliest reference year available for each location, in Figure 6 for the state of Colorado (scale 1:24,000), and for the map scales 1:24,000 and 1:62,500 for the state of California in Figure 7, respectively. Such representations are useful to identify regions that have been mapped more intensively versus those for which temporal coverage is rather sparse. Furthermore, a user is immediately informed about the earliest map sheets for a location of interest to understand the maximum time period covered by these cartographic documents. Similar representations could be created for the average number of years between editions or the time span covered by map editions of a given map scale.

**Figure 8.**
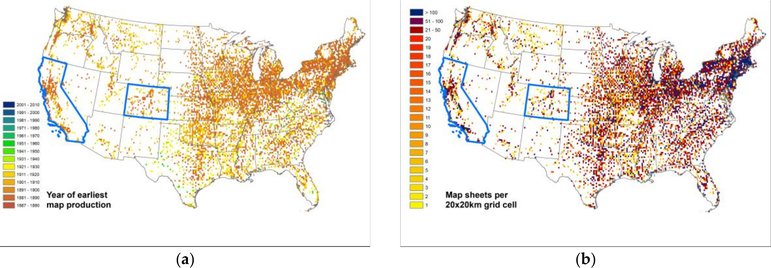
Sanborn fire insurance map archive coverage: (**a**) year of first map production per location and (**b**) number of available map sheets per location, both aggregated to grid cells of 20 km for efficient visualization. Highlighted in blue are the states of California and Colorado for comparison to the USGS map coverage shown in the previous figures.

As a second example, we visualized the spatial-temporal coverage of the Sanborn fire insurance map archive. Figure 8 shows, similar to the above examples, the year of the first map production and the number of maps produced in total per location. The comparison of these visualizations for the highlighted states of California and Colorado to the previously shown Figures 6 and 7 shows the differences in spatio-temporal coverage between the two map archives, indicating a much sparser spatial coverage of the Sanborn map archive, but extending further back in time than the USGS map archive.

#### 5.1.2. Metadata-Based Spatial-Temporal Analysis of Positional Accuracy

**Figure 9.**
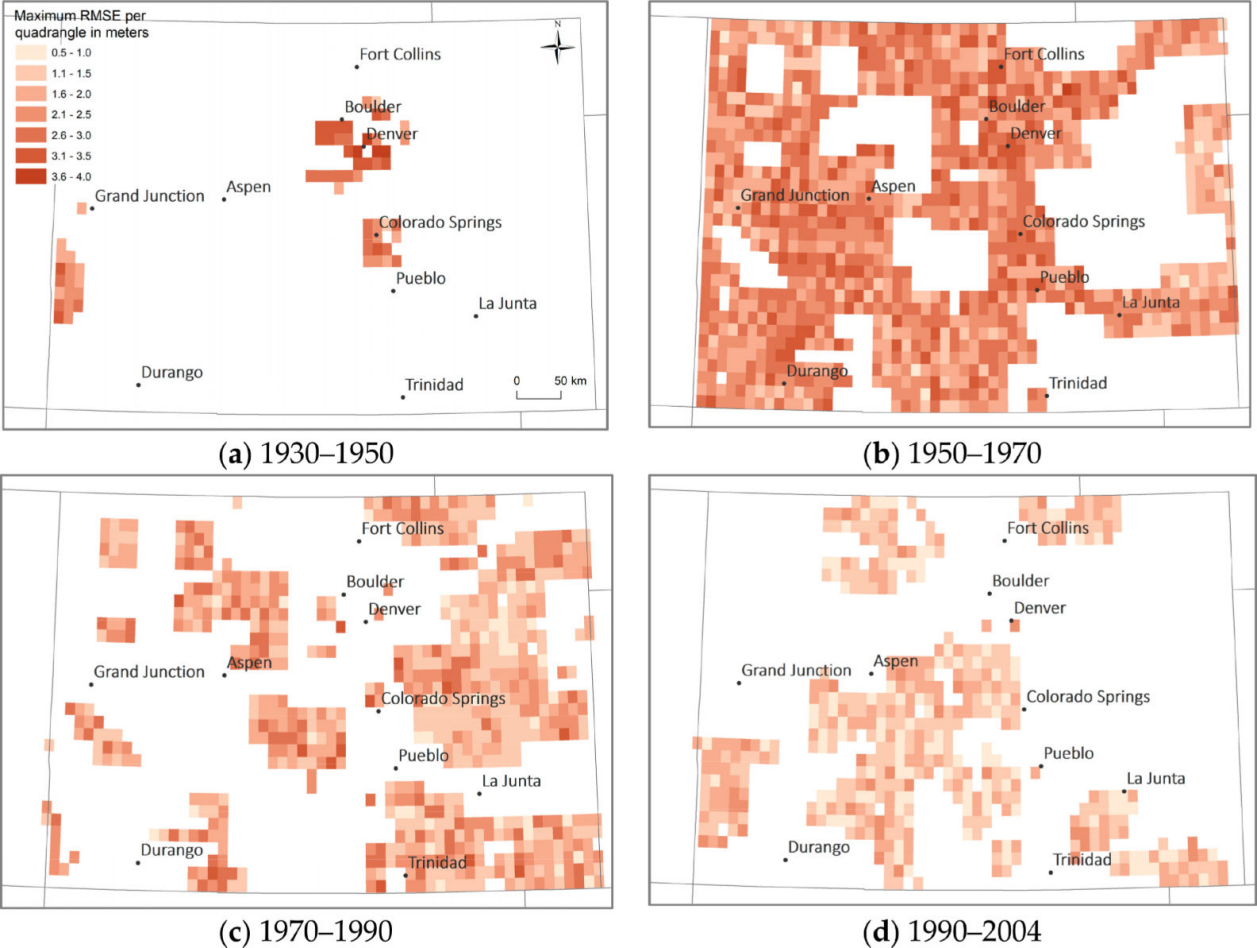
Spatio-temporal patterns of georeference accuracy of USGS topographic maps (1:24,000) in the state of Colorado (USA), for maps produced between (**a**) 1930–1950, (**b**) 1950–1970, (**c**) 1970–1990, and (**d**) 1990–2004.

To illustrate the georeference accuracy for the USGS maps of scale 1:24,000 in the state of Colorado (Figure 9) for different time periods, we visualized the maximum RMSE per quadrangle and time period. Such temporally-stratified representations are useful to examine whether the georeference accuracy is constant over time. It can be seen that the earlier years in this example show higher degrees of inaccuracy than more recent map sheets. This has important implications for the user who is interested in using maps from different points in time that may exhibit different levels of inaccuracy.

**Figure 10.**
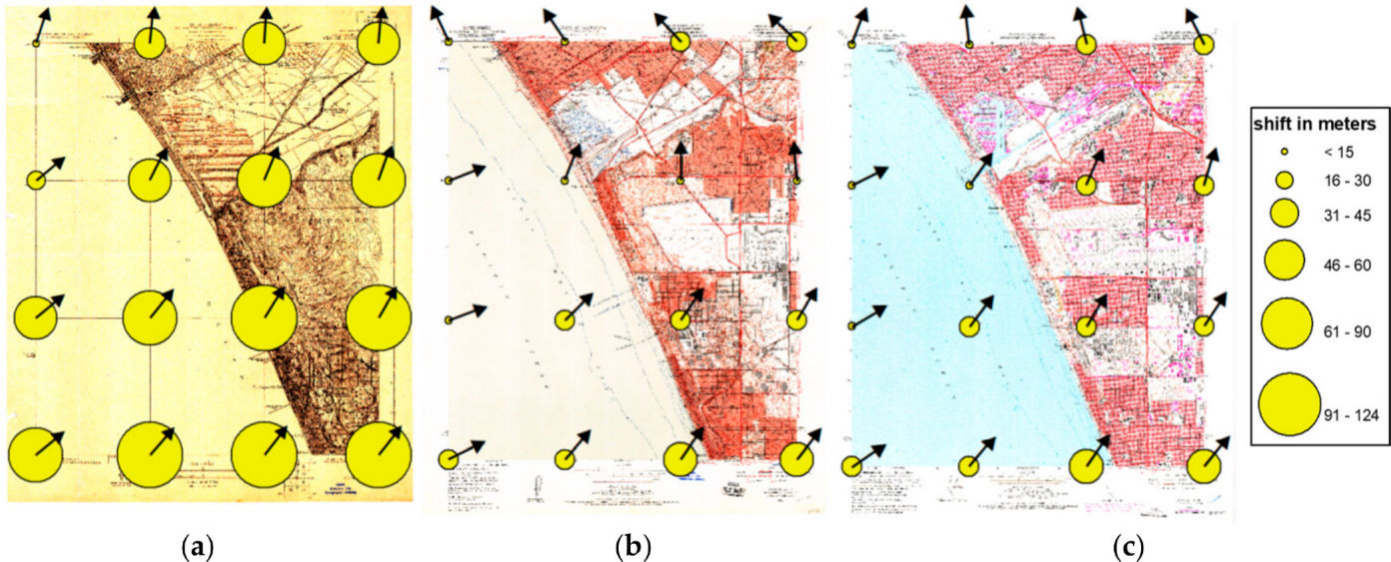
Displacement vectors at GCP locations characterizing the distortions introduced during the georeferencing of USGS topographic maps (scale 1:24,000) from Venice (California), produced in (**a**) 1923, (**b**) in 1957, and (**c**) in 1975 (from left to right).

Figure 10 shows examples of these displacement vectors visualized for individual USGS map sheets at scale 1:24,000 from Venice (California) produced in 1923, 1957, and 1975. We represent the magnitude of the local displacement by the dot area, whereas the arrow indicates the displacement angle. This example shows similar patterns across the three maps, probably reflecting non-independent distortions between the maps since earlier maps are typically used as base maps for subsequent map editions, and some local variations due to inaccuracies introduced during georeferencing of the individual map sheets.

**Figure 11.**
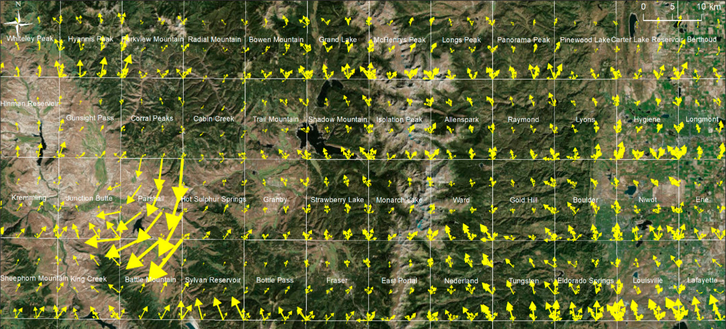
Displacement vector field at GCP locations over multiple USGS map quadrangles of scale 1:24,000, located North-west of Denver (Colorado), reflecting different types of distortions introduced to the map documents during the georeferencing process (basemap source: [50]).

Additionally, we visualized these displacement vectors as vector fields across larger areas, to identify regions, quadrangles, or individual maps of high or low positional reliability, respectively. Figure 11 shows the vector field of relative displacements for USGS maps of scale 1:24,000 for a region northwest of Denver, Colorado. Notable are the large displacement vectors in the Parshall quadrangle, indicating some anomalous map distortion, whereas the Cabin Creek quadrangle (northeast of Parshall) seems to have suffered from very slight distortions only. Such anomalous distortions as detected in the Parshall quadrangle may indicate extreme distortions in the corresponding paper map, or outliers in the GCP coordinates used for georeferencing. Multiple arrows indicate the availability of multiple map editions in given quadrangles. Such visualizations may inform map users about the heterogeneity in distortions applied to the map sheets during the georeferencing process and may indicate different degrees of positional accuracy across a given study area.

According to the USGS accuracy standards [51], a horizontal accuracy (i.e., RMSE) of <12.2 m is required for maps at a scale of 1:24,000. Whereas the georeference accuracies visualized in Figure 9 are all smaller or equal to 5 m, we found that the magnitudes of the displacement vectors shown in Figures 10 and 11 exceed the value of 12.2 m, considerably. It is important to point out that these displacement vectors may be caused by distortions in the paper map, by outliers in the GCPs, or by differences in the spatial reference systems used in the original map and for georeferencing. Thus, these displacement vectors do not represent the absolute horizontal map accuracy alone, but rather serve as measures to characterize variability in the overall distortions applied during the georeferencing across time and map sheets, and to identify anomalies, such as those shown in Figure 11, where users should be careful with respect to further information extraction from such map sheets.

### 5.2. Content-Based Analysis

#### 5.2.1. Content-Based Analysis at the Map Level

**Figure 12.**
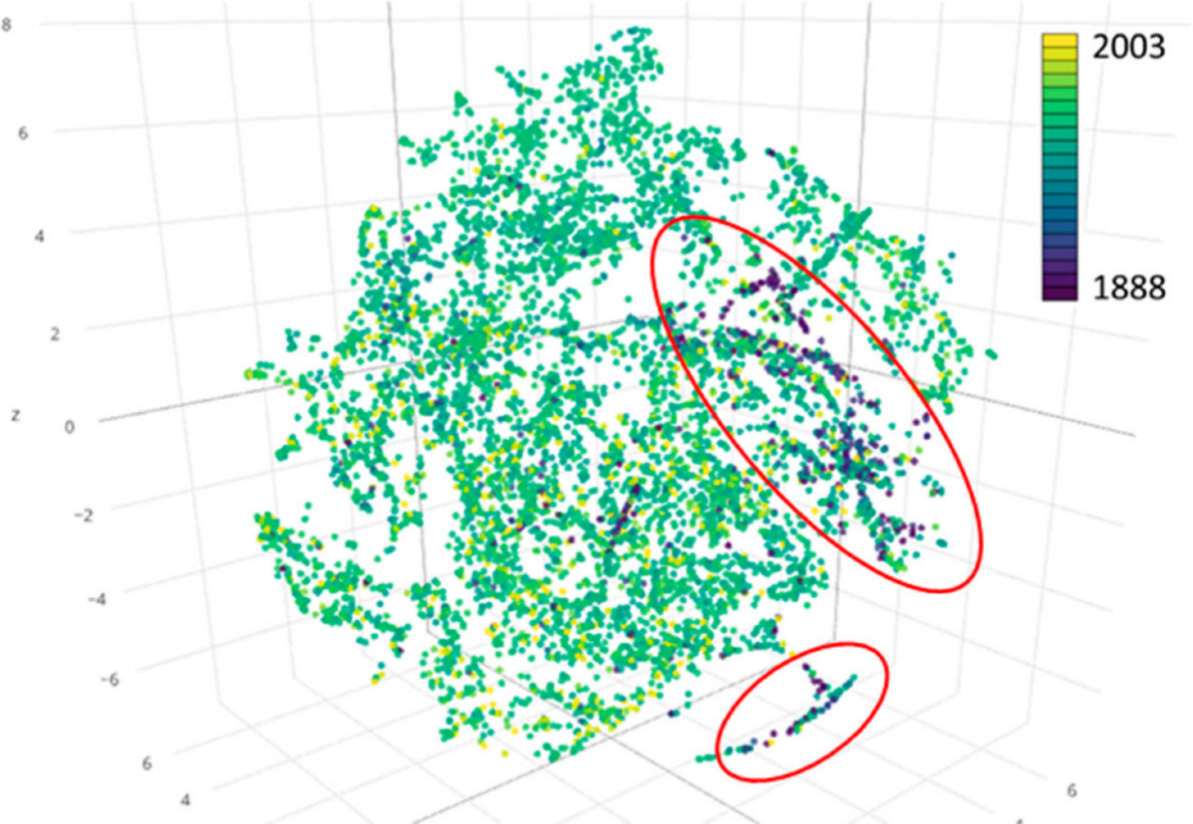
T-SNE visualization of the 6964 USGS maps in the state of Colorado in a 3D feature space based on 12-dimensional image descriptors obtained from channel-wise color moments.

Figure 12 shows the map-level image descriptors transformed into a 3D feature space for the 6964 USGS maps in the state of Colorado. We used the map reference year to color-code the points representing individual map sheets. The highlighted clusters of dark blue points indicate fundamentally different color characteristics of old maps in comparison to more recent maps represented by points colored in green-yellow tones.

**Figure 13.**
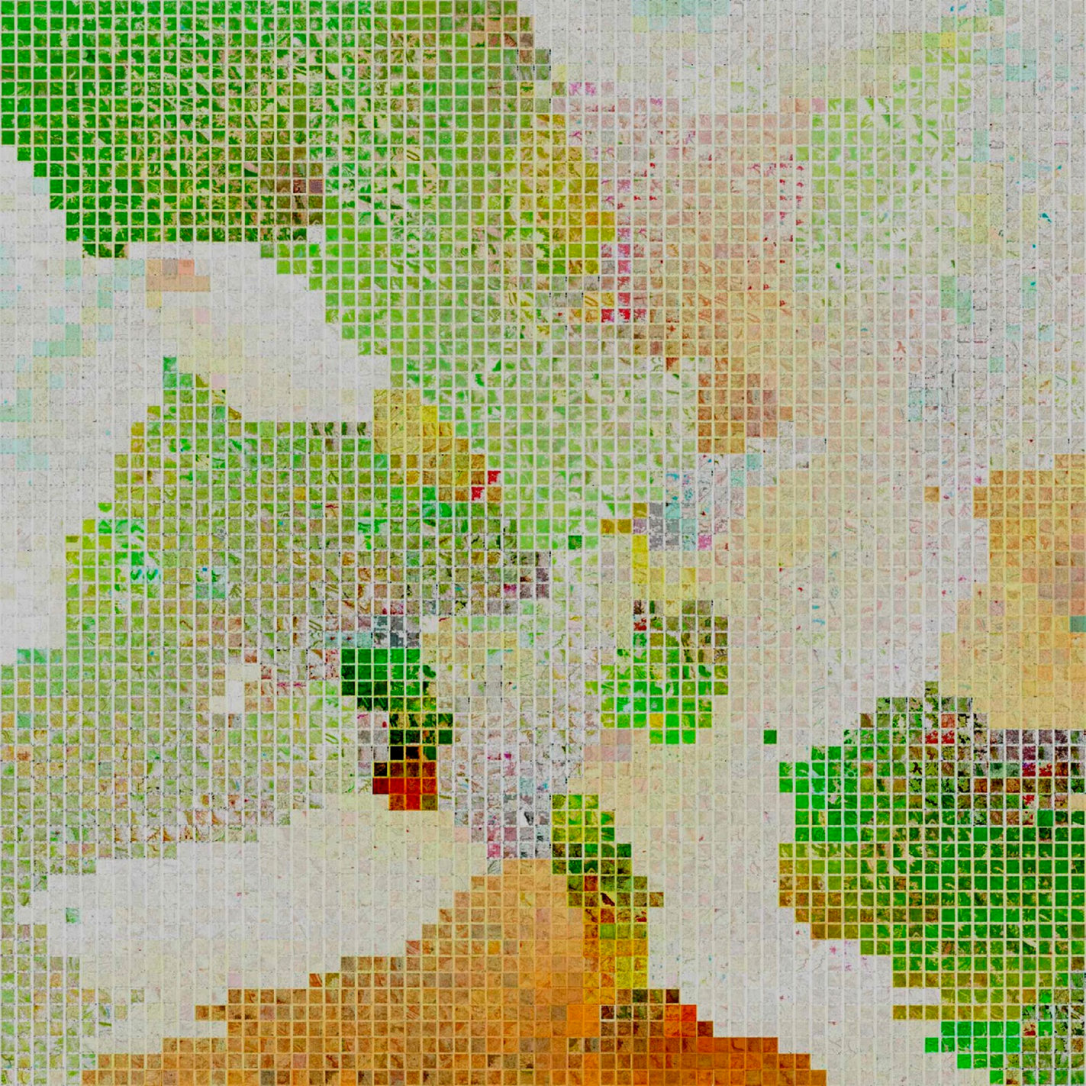
Thumbnail-based visualization of a subset of the USGS topographic maps in the state of Colorado (USA) based on a 2D transformation of the 12-dimensional image descriptor feature space using t-SNE.

In addition to color-coding the data points by the corresponding map reference year, we transformed the 12-dimensional descriptors into a 2D feature space, and visualized them using thumbnails of individual maps corresponding to each data point in Figure 12. This transformation results in an integrated visual assessment of map archives containing large numbers of map sheets. Figure 13 shows a t-SNE thumbnail visualization of a random sample (*N* = 4356) of the Colorado USGS maps in a 2D feature space. We used nearest-neighbor snapping to create a rectangular visualization. This is a very effective way to visualize the variability in map contents, such as dominating forest area proportions. It also illustrates the presence and abundance of different map designs and base color use, e.g., high contrast and saturation levels in recent maps, compared to yellow-tainted map sheets from the beginning of the 20th century centered at the bottom. The latter corresponds to the cluster of historical maps located at the bottom of the point cloud in Figure 12.

#### 5.2.2. Content-Based Analysis at Within-Map Patch Level

**Figure 14.**
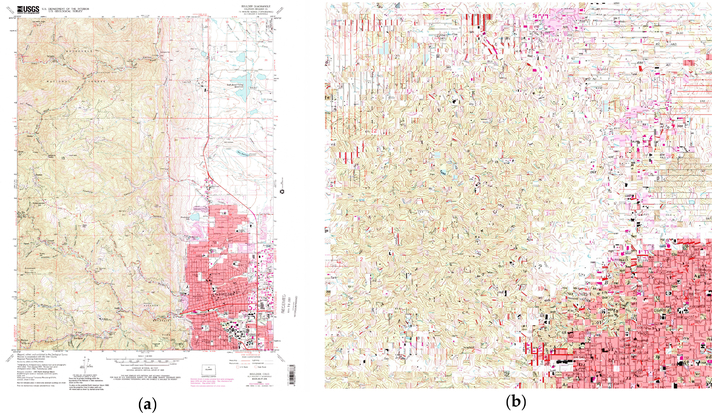
(**a**) USGS topographic map for Boulder, Colorado (1966), and (**b**) rearranged map patches according to their similarity in a raw pixel value feature space using t-SNE.

We used the t-SNE transformation of patch-level descriptors to rearrange a map document in patches based on patch similarity, as shown for an example USGS map in Figure 14a. We partitioned the clipped map content in tiles of 100 × 100 pixels, down-sampled them by a factor of four, and used the raw pixel values as input for the t-SNE transformation. This results in a 1875-dimensional feature vector per patch. We then transformed these features into a 2D-space using t-SNE in order to create a similarity-based rearrangement of the map patches (Figure 14b). This rearrangement based on raw pixel values highlights, for example, the groups of linear objects of different dominant directions, such as road objects oriented in east–west and north–south direction (Figure 14b, upper right, and upper left, respectively), or clusters of patches that contain contour lines with diffuse directional characteristics (Figure 14b, center left) The incorporation of directionality may be useful to design sampling schemes that generate training data allowing for rotation-invariant feature learning.

#### 5.2.3. Content-Based Analysis at the Cross-Map Patch Level

**Figure 15.**
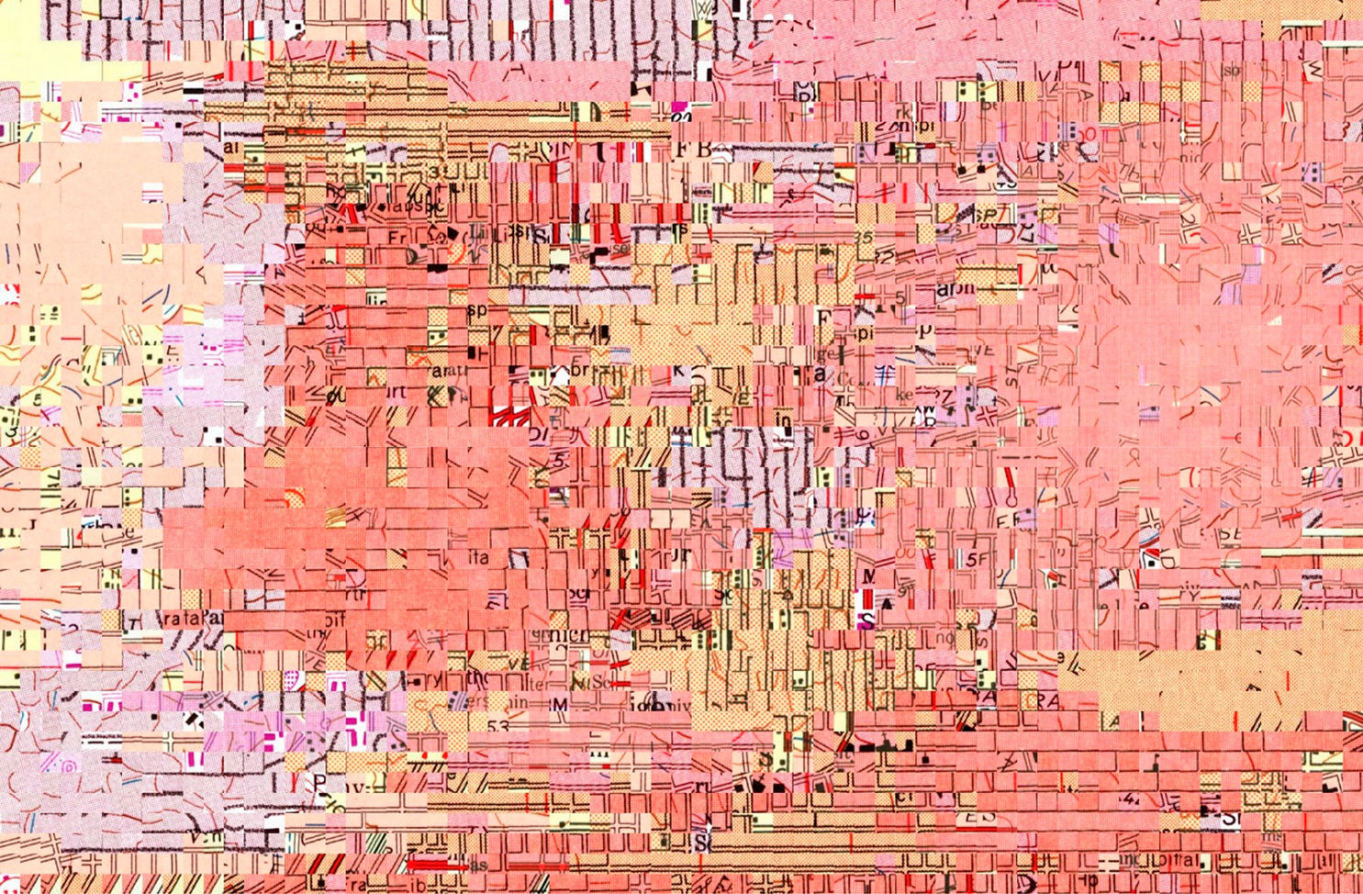
T-SNE arrangement of cross-map samples of patches likely to contain dense urban settlement symbols.

Based on ancillary data indicating the presence of dense urban settlements (see Section 4.2.3), we extracted patches that are likely to contain dense urban settlement symbols from map patches collected across 50 USGS maps (1:24,000) in the states of Colorado and California, as shown in Figure 15. This arrangement illustrates nicely the different cartographic styles that are used to represent dense urban settlements across time and map sheets, and provides valuable information useful for the design of a recognition model. Additional samples could be collected at locations where no ancillary data is available, and their content can be estimated based on descriptor similarity (i.e., patches of low Euclidean distance in the descriptor feature space) or using unsupervised or supervised classification methods.

## 6. Conclusions and Outlook

In this paper, we presented a set of methods for systematic information mining and content retrieval in large collections of cartographic documents, such as topographic map archives. These methods consist of pure metadata-based analyses, as well as content-based analyses using low-level image descriptors, such as histogram-based color moments, and dimensionality reduction methods (i.e., t-SNE). We illustrate the proposed approach by exemplary analyses of the USGS topographic map archive and the Sanborn fire insurance map collection. Our approach can be used to explore and compare spatio-temporal coverage of these archives, the variability of positional accuracy, and differences in content of the map documents based on visual-analytical tools. These content-based map mining methods are inspired by image information mining systems implemented for remote sensing data archives.

More specifically, analysts aiming to develop information extraction methods from large map archives can benefit from the proposed methods as follows:

### Spatio-temporal coverage analysis:

- Estimation of the spatio-temporal coverage of the extracted data; and
- Guidance for the design of the training data collection, to ensure the collection of balanced and representative training data across the spatio-temporal domain.

### Spatio-temporal analysis of spatial accuracy:

- Estimating the spatial accuracy of the extracted data; and
- Excluding map sheets of potential low spatial accuracy to ensure high degrees of spatial alignment of map and ancillary data used for training data collection and, thus, to reduce noise in the collected training data

### Content-based image analysis:

- Assessing the variations in map content as a fundamental step in order to choose adequate information extraction methods capable of handling data of the given variability and to create representative training data accounting for such variations.

The presented methods have been tested and proven useful as preliminary steps to facilitate the design and implementation of information extraction methods from historical maps, e.g., regarding the choice of training areas and classification methods [34]. Further work will include the incorporation of suitable image descriptors accounting for textural information contained in map documents. Additionally, the benefit of indexing techniques based on image descriptors will be tested in a prototype map mining framework, facilitating the retrieval of similar map sheets in large map archives. Moreover, these efforts will contribute to the design of adequate sampling methods to generate large amounts of representative training data for large-scale information extraction methods from historical map archives based on deep-learning methods.

Such large-scale extraction of retrospective geographical information from historical map archives will contribute to create analysis-ready geospatial data for time periods prior to the era of digital cartography and, thus, help to better understand the spatial-temporal evolution of human settlements, transportation infrastructure, forest coverage, or hydrographic features and their interactions with social and socio-economic phenomena over long periods of time. Such knowledge may be used to support and improve predictive land cover change models, and constitutes a valuable information base for decision making for planning or conservation purposes.

Similarly to web-based data storage and processing platforms for remote sensing data [52–54], adequate computational infrastructure will be required for effective processing of large volume map archives. The USGS data used in this study are accessed through a web storage service. We expect that, in the near future, additional map archives will be made available using similar web-based storage services that will facilitate the direct incorporation of the data into information extraction processes (e.g., based on deep learning) implemented in cloud-computing platforms at reasonable computational performance, and without previous manual and time-consuming data download.

The discussed content-based image analysis can be extended to most types of map archives as presented. The described metadata-based methods have the potential to be adapted to other existing map archives if metadata and georeference information is available in ways similar to the archives presented in this work. This study aims to raise awareness of the importance of a-priori knowledge of large spatial data archives before using the data for information extraction purposes and help to anticipate the potential challenges involved. Such systematic mining approaches of relevant information about map archives help to inform and educate the user community on critical aspects of data availability, quality, and spatio-temporal coverage.

In conclusion, this work demonstrates how state-of-the-art data analysis and information extraction methods are not only useful to handle and analyze large amounts of contemporary or real-time streaming data, but also provide computational infrastructure suitable for processing historical geospatial data.
